# Thromboses des artères rénales révélant un syndrome des anticorps antiphospholipides

**DOI:** 10.11604/pamj.2018.31.150.15677

**Published:** 2018-10-30

**Authors:** Wafaa Arache, Abdelali Bahadi, Driss El Kabbaj

**Affiliations:** 1 Service de Néphrologie, Dialyse et Transplantation Rénale, Hôpital Militaire d’Instruction Mohammed V, Rabat, Maroc

**Keywords:** Le syndrome des anticorps antiphospholipides, thromboses vasculaires, thrombotique artériel et/ou veineux, Antiphospholipid antibody syndrome, vasculars thrombosis, arterial and/or venous thromboembolic

## Abstract

Le syndrome des anticorps antiphospholipides est une thrombophilie définie par l'association d'un événement clinique thrombotique artériel et/ou veineux, ou obstétrical à la présence durable d'anticorps antiphospholipides. Nous rapportons le cas d'une jeune patiente admise pour une insuffisance rénale aiguë sur thrombose bilatérale des artères rénales et chez qui le bilan immunologique a révélé la présence d'anticoagulant circulant de type lupique faisant retenir le diagnostic d'anticorps antiphospholipides. La prise en charge rapide de la thrombose des artères rénales par angioplastie a permis la récupération d'une fonction rénale normale.

## Abstract (en)

Antiphospholipid antibody syndrome is a thrombophilia characterized by the association of a clinical or obstetric arterial and/or venous thromboembolic event with persistent antiphospholipid antibodies. We here report the case of a young patient admitted with acute renal failure associated with bilateral renal artery thrombosis. Immunology tests showed lupus-like circulating anticoagulant on the basis of which the diagnosis of antiphospholipid antibodies was retained. Early angioplasty was performed enabling renal function recovery.

## Introduction

Le syndrome des anticorps antiphospholipides (SAPL) est une affection systémique individualisée en 1968 [1], et dont la symptomatologie clinique est dominée par la survenue de thromboses qui peuvent toucher tout l'arbre vasculaire artériel et veineux qu'il faudrait repérer au plus tôt afin de limiter ses conséquences et instaurer un traitement anti thrombotique prophylactique à long terme.

## Patient et observation

**Figure 1:**
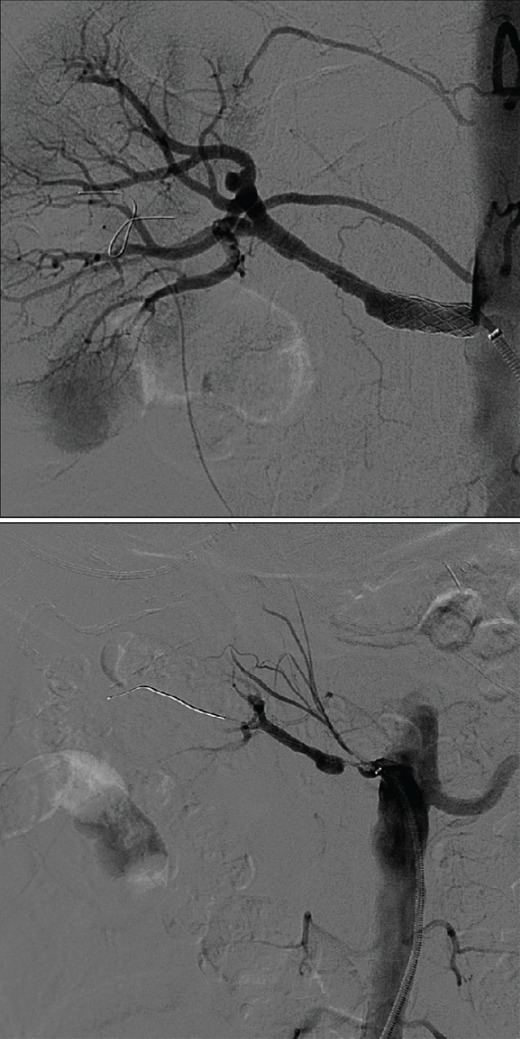
angioplastie d’une artère rénale droite

Il s'agissait d'une patiente âgée de 17 ans, originaire de la Guinée équatoriale, sans antécédents de diabète, ni d'hypertension artérielle, ni de prise de toxiques. Elle était admise pour une hypertension artérielle sévère dans un contexte d'encéphalopathie hypertensive révélée par des convulsions, la patiente rapportait une baisse bilatérale de l'acuité visuelle sans dyspnée, ni palpitations, ni sueurs. L'examen clinique objectivait une TA à 220/160 mmHg aux deux bras, tous les pouls périphériques étaient présents, avec à la bandelette urinaire une hématurie à trois croix et une protéinurie à une croix. Le bilan biologique trouvait une insuffisance rénale avec une créatinine à 34mg/l et une urée à 1.5g/l sans troubles électrolytiques; une microangiopathie thrombotique avec une hémoglobine à 11,4g/dl; un taux de plaquettes à 47000elmts/mm^3^ et des LDH à 5240 UI/l sans hyperleucocytose. Le bilan inflammatoire était normal, la microalbuminurie de 24 H était de 0,9g/jour. Le doppler des artères rénales objectivait des reins de taille normale, une aorte libre avec un tracé démodulé sur les troncs des artères rénales avec augmentation des index de résistances; l'angio-scanner a objectivé un rétrécissement moniliforme des troncs des deux artères rénales très serré avec des artères interlobulaires à paroi irrégulière sans nodule surrénalien, des reins de taille normale avec un aspect perméable des veines rénales et de la VCI. L'artériographie a objectivé une thrombose complète des deux artères rénales, une angioplastie était réalisée avec mise en place d'un stent rénal droit (Figure 1).

La scintigraphie rénale au DMSA objectivait un rein droit assurant 83% de la fonction rénale et un rein gauche assurant 17% avec hypofixation au niveau du rein gauche et du pole supérieur du rein droit.

**Sur le plan cardiaque:** l'échographie cardiaque montrait une hypertrophie ventriculaire gauche concentrique, une hypokinésie globale, une fraction d'éjection à 51% avec une oreillette gauche dilatée et des cavités droites normales. La patiente a été mise sous Amlodipine* 10 mg / jour, Sectral * 400 mg / jour en deux prises, Fludex* LP (1 comprimé par jour), Hyperium* 1 mg (2 comprimés par jour et Lasilix* 40 mg/jour). Sur le plan ophtalmologique, le fond d'œil a révélé des vaisseaux rétiniens grêles avec une occlusion artériolaire et des exsudats péri papillaires et maculaires (rétinopathie hypertensive stade 2 et 3) l'angiographie à la fluorescéine montrait des exsudats cercinés avec des zones d'ischémie focales et engainement vasculaire.

**Sur le plan neurologique:** l'imagerie par résonnance magnétique cérébrale montrait des anomalies du signal vasculaire en sus tentoriel, l'électro-encéphalogramme objectivait une souffrance cérébrale avec des anomalies épileptiques diffuses posant le diagnostic d'encéphalopathie hypertensive mise sous dépakine chrono* (1 comprimé 2 fois par jour).

**Le bilan immunologique:** à la recherche de vascularite a objectivé des anticorps antinucléaires positifs (1/80), Ac anti ADN négatifs, anti nucléaires solubles: Anti-SSA, Anti-SSB, anti-Sm, Anti-Scl70, Anti-Jo1, Anti CENP-B, Ribosome P Protéines étaient revenus tous négatifs. Les anticorps anticardiolipineIgG et IgM (sur Alegria par Elisa SMC) et les auto-anticorps anti Beta 2 glycoprotéine I, IgG étaient positifs. Par ailleurs, le dosage de l'homocystéine et la recherche d'anticoagulant circulant de type lupique étaient revenus franchement positifs. Le bilan de thrombophilie avait objectivé des taux normaux de l'antithrombine II et un taux bas de la protéine S. Après discussion du dossier avec les angiologues, le diagnostic du SAPL a été retenu avec mise sous anticoagulants à base d'anti vitamine K (sous surveillance de L'INR: International Normalized Ratio). L'évolution après le geste endovasculaire et le contrôle de la poussée hypertensive était marquées par la normalisation de la fonction rénale.

## Discussion

Le SAPL rendrait compte de 15 à 20% des épisodes de thromboses veineuses profondes et d'un tiers des accidents vasculaires cérébraux survenant avant l'âge de 50 ans [2, 3]. Classiquement, le SAPL peut être primitif ou secondaire à des maladies systémiques (en premier lieu le lupus érythémateux disséminé, mais également la polyarthrite rhumatoïde, la sclérodermie, le syndrome de Sjögren…), à des infections diverses (VIH, hépatite C), des prises médicamenteuses voire à des maladies lymphoprolifératives [4]. La pathogénie de ce syndrome n'est pas complètement connue, les anticorps dits «antiphospholipides » ont la particularité de se lier à des récepteurs à la surface des membranes cellulaires, ce qui entraine une activation endothéliale vasculaire avec une sécrétion de cytokines pro-inflammatoires de radicaux libres et de molécules d'adhésion. Ces anticorps inhibent l'action des protéines telles que la béta glycoprotéine, aboutissant in fine à un état pro-coagulant [5]. Le diagnostic positif du SAPL est basé sur des critères clinico-biologiques [6, 7], le diagnostic positif est retenu devant l'association d'au moins un critère clinique et un critère biologique (Tableau 1). Chez les patients présentant simultanément des anticoagulants circulants de type lupique, des anticorps béta glycoprotéine I et des anticorps anticardiolipides, le risque de survenue d'un événement thrombotique est plus fréquent dans les populations de patients avec simple ou double positivité, y compris en cas de positivité des anticorps décelée avant tout événement clinique [8].

**Tableau 1:** critères de classification du syndrome des antiphospholipides

Critères cliniques	Manifestations
**Thrombose**	Un ou plusieurs épisodes symptomatiques de thrombose artérielle et veineuse dans n’importe quel tissu ou organe.
**Manifestation obstétricale**	Une ou plusieurs morts fœtales inexpliquées avec fœtus morphologiquement normal à partir de la 10ème semaine de gestation ou au-delà ou une ou plusieurs naissances prématurés d’un nouveau-né morphologiquement normal avant la 34ème semaine de gestation liées à une éclampsie grave ou des signes reconnus d’insuffisance placentaire ou au moins 3 avortements spontanés consécutifs avant la 10ème semaine de gestation sans cause anatomique ou hormonale maternelle et sans cause chromosomique parentale reconnue.
**Critères biologiques**	Présence au minimum à 2 reprises espacées de 12 semaines d’intervalle d’un anticoagulant circulant de type lupique ou anticorps anticardiolipides d’isotype Ig G et Ig M dans le sérum ou le plasma avec un titre moyen ou élevé (> 40GPL ou MPL ou > 99^e^ percentile) avec des mesures par ELISA standardisé, ou des Anticorps anti b2 glycoprotéines I d’isotype IgG et/ou Ig M dans le sérum ou le plasma avec un titre moyen ou élevé (> 40GPL ou MPL ou > 99^e^ percentile)

**Figure 2:**
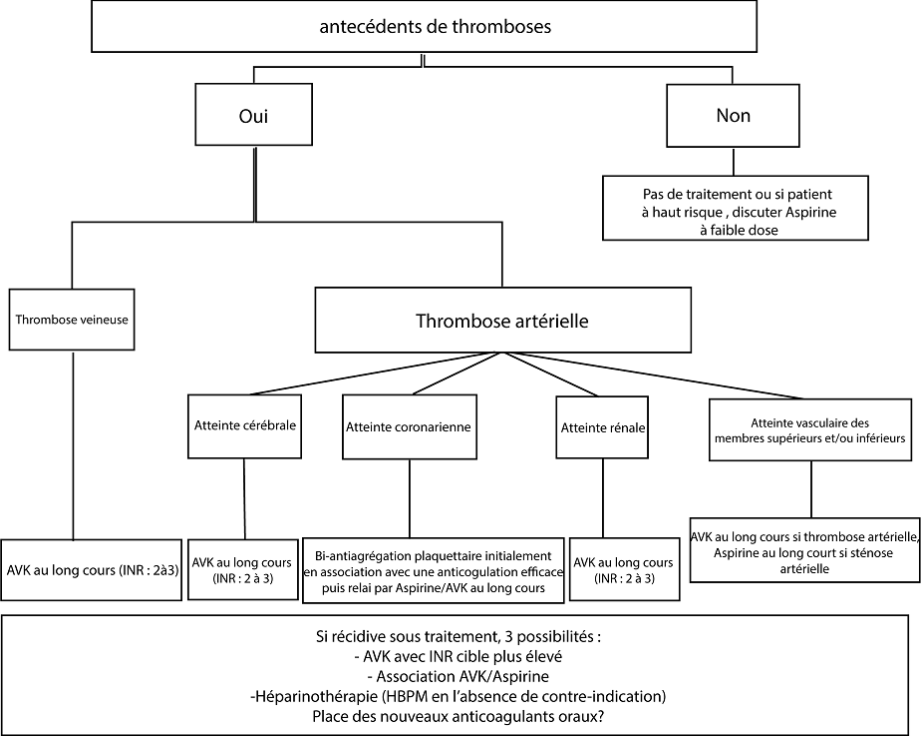
gestion du traitement antithrombotique au long cours du SAPL

L'atteinte rénale au cours du SAPL est caractérisée par une néphropathie vasculaire pouvant toucher toutes les structures vasculaires rénales dont on décrit deux types: une forme artérielle (proximale et/ou distale, aiguë et/ ou chronique) et une forme veineuse. La forme artérielle proximale est définie par la présence d'une sténose ou d'une thrombose dans les artères rénales de gros calibre (tableau d'infarctus rénal). La néphropathie artérielle distale est l'atteinte rénale la plus fréquente, le diagnostic est fait sur la biopsie rénale objectivant une congestion dans les capillaires glomérulaires avec présence d'espaces clairs sous-endothéliaux et de doubles contours des parois des capillaires glomérulaires [9]. La forme veineuse est plus rare que la forme artérielle; elle se caractérise essentiellement par une thrombose de la veine rénale ou des veines en amont. Le diagnostic est habituellement donné par l'imagerie. La recherche d'une extension cave, d'une hémorragie bilatérale des surrénales ou d'une embolie pulmonaire doit être systématique. La thérapeutique se base comme chez notre patiente sur une anticoagulation plus ou moins associée à une antiagrégation à long terme en fonction des événements cliniques (Figure 2). Simultanément, le contrôle strict des facteurs de risque cardiovasculaire classiques ne doit pas être négligé. Différents algorithmes et recommandations ont été proposés dans la littérature pour le traitement du SAPL [10].

## Conclusion

Le syndrome des antiphospholipides est une thrombophilie acquise caractérisée par l'association de manifestations clinico-biologiques. L'atteinte rénale est fréquente et sévère avec des lésions de néphropathie associée au SAPL qui sont aujourd'hui bien décrites. Cette atteinte conduit de façon non exceptionnelle à une insuffisance rénale chronique terminale en l'absence de prise en charge codifiée.

## Conflits d’intérêts

Les auteurs ne déclarent aucun conflit d’intérêts.
